# Use of the Ketogenic Diet to Treat Intractable Epilepsy in Mitochondrial Disorders

**DOI:** 10.3390/jcm6060056

**Published:** 2017-05-26

**Authors:** Eleni Paleologou, Naila Ismayilova, Maria Kinali

**Affiliations:** Chelsea and Westmister Hospital, 369 Fulham Road, Chelsea, London SW10 9NH, UK; elenipal13@yahoo.com (E.P.); ismayilova@doctors.org.uk (N.I.)

**Keywords:** ketogenic diet, mitochondrial disorders, intractable epilepsy, treatment

## Abstract

Mitochondrial disorders are a clinically heterogeneous group of disorders that are caused by defects in the respiratory chain, the metabolic pathway of the adenosine tri-phosphate (ATP) production system. Epilepsy is a common and important feature of these disorders and its management can be challenging. Epileptic seizures in the context of mitochondrial disease are usually treated with conventional anti-epileptic medication, apart from valproic acid. However, in accordance with the treatment of intractable epilepsy where there are limited treatment options, the ketogenic diet (KD) has been considered as an alternative therapy. The use of the KD and its more palatable formulations has shown promising results. It is especially indicated and effective in the treatment of mitochondrial disorders due to complex **I** deficiency. Further research into the mechanism of action and the neuroprotective properties of the KD will allow more targeted therapeutic strategies and thus optimize the treatment of both epilepsy in the context of mitochondrial disorders but also in other neurodegenerative disorders.

## 1. Introduction in Mitochondrial Disorders and Mitochondrial Epilepsy

Mitochondrial disorders are a clinically heterogeneous group of disorders arising from defects in the respiratory chain, the metabolic pathway of the mitochondrial adenosine tri-phosphate (ATP) production system via oxidative phosphorylation (OXPHOS). ATP is commonly referred to as the “molecular energy unit” of cells providing the energy source for metabolic functions. Mitochondrial disorders can affect any tissue or organ, but those most affected are those with the highest energy demands, such as the central nervous system, skeletal and cardiac muscles, kidney, liver and endocrine system.

Although mitochondrial diseases have traditionally been considered rare diseases, epidemiological studies suggest otherwise. A United Kingdom (UK) study in 2008 showed that 9.2 people in 100,000 of working age (between 16 and 65 years of age) had a clinically manifested mitochondrial DNA (mtDNA) disease. A further 16.5 children and adults younger than retirement age per 100,000 were at risk of developing one [1]. Other studies quote a minimum prevalence of at least one in 10,000 children and adults, with one in 200 children with a congenital mitochondrial DNA mutation [2]. Bearing in mind that several mtDNA mutations are being discovered each year, these numbers likely underestimate the true prevalence.

Diagnosing mitochondrial disorders can be challenging given their extremely broad clinical spectrum and the lack of consistent phenotype-genotype correlations [3]. Tests that would help with diagnosis range from simple laboratory blood tests, electrocardiograms, genetic testing, hearing and ophthalmology assessments, brain imaging, lumbar puncture, and muscle biopsy, while new tests are being evaluated to facilitate the diagnosis. 

A complete laboratory diagnostic work-up, and evaluation of the results in the context of the clinical phenotype and family history are preferred. Elevated blood or cerebrospinal fluid (CSF) lactate can be suggestive of mitochondrial disease, but their respective normality cannot exclude this diagnosis. Likewise, electrocardiograms (ECG) can show heart block in Kearns-Sayre syndrome (KSS) or mitochondrial encephalopathy, lactic acidosis and stroke-like episodes (MELAS), or pre-excitation in MELAS or myoclonus epilepsy with ragged red fibres (MERFF), but a normal ECG does not exclude the diagnosis. In addition, brain magnetic resonance imaging (MRI) can reveal basal ganglia calcification or atrophy or involvement of the occipital lobes, the thalamus and inferior olivary nuclei. Magnetic resonance spectroscopy can demonstrate elevated lactate in the brain, whereas positron emission tomography (PET) scanning can suggest lowered ATP production. While these tests can be suggestive of mitochondrial disorders they are neither sensitive nor specific [3].

Ultimately, the biochemical examination of a skeletal muscle biopsy to evaluate the functional state of mitochondria and to look for the characteristic ultrastructural abnormalities seen in mitochondrial disease remains the optimal method of diagnosing mitochondrial disorders, even in the absence of myopathy. Biopsies provide material for respiratory chain enzyme assays to look for characteristic abnormalities like the presence of ragged red fibres (RRF) with abnormal mitochondria in MERFF, KSS or MELAS using the Gomori trichrome stain or succinate dehydrogenase (SDH)-reactive vessels, and the preservation of cytochrome c oxidase (COX) staining in RRFs often seen in MELAS [3,4]. Skeletal muscle biopsies can also be analyzed for mtDNA. Histochemical stains for mitochondrial enzymes can reveal specific enzyme abnormalities and blue native polyacrylamide gel electrophoresis has been shown to be very useful in analyzing the function of individual enzyme complexes and detecting assembly defects [5]. It is important to bear in mind that on rare occasions the histological changes described may be absent.

Novel biomarkers, like fibroblast growth factor 21 (FGF-21) which has been shown to be a sensitive and specific biomarker for mitochondrial disease affecting skeletal muscles, may play a more crucial role in the future. FGF-21 has also been shown to be useful for monitoring of disease progression and to assess the effect of therapeutic interventions, but is not yet commercially available [6]. There is a urine test currently only available in research laboratories for the diagnosis of progressive external ophthalmoplegia (PEO) and KSS, which would revolutionize the way we diagnose these conditions if made commercially available [7].

The metabolic pathway that generates ATP via oxidative phosphorylation is regulated both by mtDNA, which is maternally inherited, as well as nuclear DNA (nDNA), which is inherited in an autosomal or X-linked manner. Mutations in either of these genomes can result in mitochondrial disorders. mtDNA diseases, therefore, assume a complex pattern of inheritance, which includes maternal, X-linked, recessive and dominant transmission modes [2,3]. Over 270 mutations in mtDNA have been described with mutations in the mitochondrial transfer ribonucleic acid (tRNA) genes being particularly common. Epilepsy remains a common feature in conditions that result from these mutations and notably status epilepticus, which can be the presenting feature. Polymerase Gamma (POLG) is a gene that codes for the catalytic subunit of the mitochondrial DNA polymerase gamma and is crucial for replicating mitochondrial DNA and for DNA repair. Although many mitochondrial nuclear gene defects can cause epilepsy, POLG mutations have been studied the most [2,3]. 

With the rapid advances in the field of genetics and the emergence of next generation sequencing, new genes are continuously discovered as linked to mitochondrial disease. This technique allows sequencing of multiple candidate genes at the same time, increasing the diagnostic yield, but is still mainly a research tool [8]. Moreover, whole mtDNA sequencing of blood samples is now possible in some diagnostic laboratories and could be a useful screen for patients whose family history suggests maternal inheritance. Patients with clinical suspicion of Alpers-Huttenlocher syndrome, sensory ataxic neuropathy dysarthria, ophathalmoplegia (SANDO), or PEO should be screened for the POLG mutations [7].

To date, there is no cure for mitochondrial disorders, but treatment aims at improving function of the respiratory chain. A “cocktail” of supplements is used by clinicians which includes Co-enzyme Q10 (CoQ10), alpha-lipoic acid, riboflavin, folic acid, creatine monohydrate, thiamin, niacin, pantothenic acid, vitamins B12, C, E, biotin and nitric oxide precursors. Although these supplements are safe and well tolerated, their efficacy is limited [3]. A Cochrane review from 2012 did not find enough evidence to support the use of any treatment in mitochondrial disease [9]. However, there are examples where specific treatment can prove useful, like Co-enzyme Q10 (CoQ10) supplementation in primary CoQ10 deficiency or idebenone, a CoQ analogue and cofactor for NADPH dehydrogenase, in patients with Leber’s hereditary optic neuropathy (LHON) [3]. In addition, aerobic exercise is known to improve mitochondrial biogenesis and lead to mitochondrial proliferation [3]. Lastly, treatments specifically aimed at the various symptoms of mitochondrial disease, like physiotherapy for hypotonia or cochlear implants for hearing loss, also form part of the management of MD.

The central nervous system (CNS) is the second organ most frequently affected by mitochondrial diseases. Epilepsy is a common feature [3,10], although preceded by other clinical presentations in most cases [11]. In fact, mitochondrial dysfunction has been shown to both generate seizures as well as result in neuronal cell death [12]. Seizures can manifest at any age and be the presenting feature of an underlying biochemical defect. Despite a genetic aetiology, they may even occur in the absence of a clear family history. The clinical spectrum of epilepsy from mitochondrial respiratory chain defects is variable, ranging from early-onset Ohtahara syndrome to late-onset Landau-Kleffner syndrome, as well as focal and generalized types of epilepsy [13]. Although there are no data on the exact prevalence of epilepsy in mitochondrial disorders, seizures are more common in some mitochondrial disorders than in others [10]. For example, in children with mitochondrial disorders the reported prevalence of seizures ranged between 35% and 61% [12]. Whittaker et al., found an overall prevalence of epilepsy of 23.1% in a prospective cohort of 182 adults with mitochondrial disease followed up for 7 years [14].

Mitochondrial disorders that occur due to defects in the respiratory chain usually have epilepsy as part of their clinical phenotype. Moreover, the mitochondrial syndromes with epilepsy as a leading clinical feature include MERFF, Alpers-Huttenlocher syndrome, Leigh syndrome, myoclonic epilepsy myopathy sensory ataxia (MEMSA), MELAS and others [12]. The seizures most commonly encountered in these disorders are myoclonic and focal, with a predilection to occipital lobes (at least initially), but can be generalized tonic–clonic seizures and status epilepticus [2,12]. In MELAS specifically, the seizures encountered are often associated with migraine-like headache and affected individuals frequently present in status epilepticus. They also commonly present with symptomatic focal seizures secondary to strokes. When myoclonus is present, it is less severe and more infrequent than in MERFF [2]. Myoclonus, which can be epileptic or non-epileptic, can be seen in all types of mitochondrial disease. It can be practically constant or intermittent, photosensitive or intensified by actions like writing.

There is no single anti-epileptic drug (AED) specifically indicated in mitochondrial disease. In addition, a lot of the commonly used antiepileptic drugs are mitochondrial toxic and could worsen the condition or even prove fatal [15]. While most of the seizure types (including generalized tonic-clonic seizures and status epilepticus) in mitochondrial disease are generally amenable to traditional anti-epileptic treatment, myoclonus often remains refractory to medical treatment and is likely to be progressive. This could suggest a non-epileptic origin of myoclonus.

Usually, a combination of AEDs (including benzodiazepines) is necessary to achieve the best control of the seizures. Aggressive treatment is usually advised to try and prevent secondary damage. In the context of mitochondrial disease, it is crucial to establish whether a disorder is due to a nuclear-encoded POLG mutation, such as Alpers-Huttenlocher syndrome, as sodium valproate is absolutely contraindicated in patients with this disease [15]. Sodium valproate could accelerate a tendency to liver failure and expedite death. It is well known that numerous AEDs disrupt mitochondrial function by interfering with the respiratory chain and can be toxic in mitochondrial disorders. However, the precise mechanism of toxicity is not fully understood and there is considerable individual variability on how well these drugs are tolerated. AEDs that are known to be toxic in mitochondrial disorders, but less than sodium valproate, include phenobarbital, carbamazepine, phenytoin, oxcarbazepine and ethosuximide [15]. Mitochondrial dysfunction leads to disruption in calcium homeostasis, which can increase neuronal excitability causing seizures. Levetiracetam is one of the most effective AEDs in mitochondrial epilepsy as it modulates intracellular calcium influx [16].

It is also important to note that drug interventions that target a single biological pathway will only help the specific individuals where that drug’s mechanism of action is relevant to their disorder. Since we know that epilepsy in mitochondrial disorders is complex and includes numerous types of seizures, it is no surprise that a single AED almost never results in seizure control. Challenges in effectively controlling the epilepsy in mitochondrial disorders have prompted the use of the ketogenic diet (KD) either in first-line or in refractory epilepsy.

## 2. Pathogenesis of Mitochondrial Epilepsy

**Figure 1:**
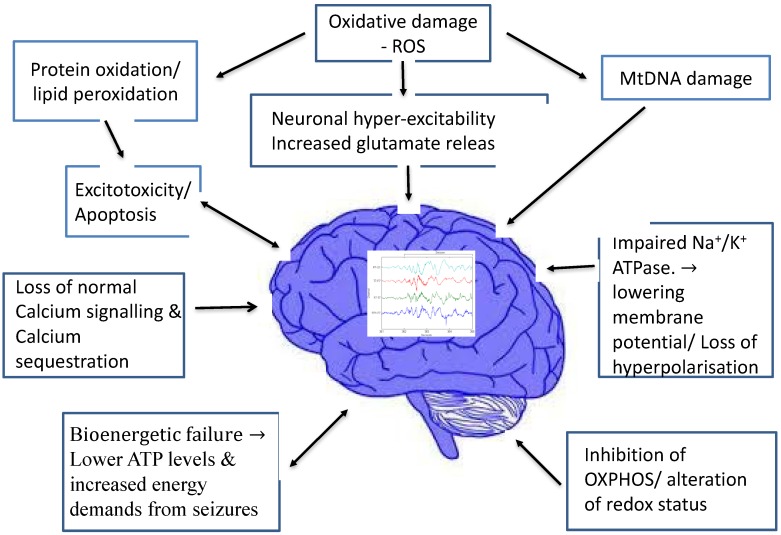
Potential mechanisms of epileptogenesis in mitochondrial disease. (ROS: Reactive Oxygen Species; ATP: Adenosine Tri-Phosphate; Na^+^: Sodium; K^+^: Potassium; MtDNA: mitochondrial DNA; OXPHOS: oxidative phosphorylation).

Mitochondria are organelles commonly denoted as the “power house of the cell” where ATP is generated via oxidative phosphorylation, mainly by using pyruvate derived from glycolysis. Ketone bodies generated by fatty acid oxidation can serve as alternative metabolites for aerobic energy production [3,17]. Figure 1 summarizes the various proposed mechanisms through which mitochondrial dysfunction is linked to epileptogenesis.

Increasingly, mitochondrial dysfunction has been recognized as a common mechanism underlying many neurological disorders [18]. Since neurons have high-energy demands and no significant capacity to regenerate, they are particularly vulnerable to mitochondrial dysfunction. Mitochondria, in turn, are the primary site of reactive oxygen species (ROS) making them particularly vulnerable to oxidative damage [19]. The latter could contribute to neuronal hyper-excitability and seizures. Mitochondrial dysfunction may also be an important cause of therapy-resistant types of severe epilepsy. Studies have confirmed the disruption of the anti-oxidant protection system and increased production of ROS in patients with epilepsy. Animal seizure models have also shown neuroprotective effects following both endogenous and exogenous anti-oxidants [20]. 

It is known that mitochondrial impairment occurs acutely, due to precipitating injuries such as status epilepticus. However, mitochondrial dysfunction is also implicated in epileptogenesis and acquired, chronic, epilepsy, like that seen in temporal lobe epilepsy (TLE) [19,21]. Experimental models of TLE have suggested mitochondrial dysfunction and oxidative stress may play a crucial role in epileptogenesis, through various mechanisms which include mitochondrial DNA damage, protein oxidation, lipid peroxidation and changes in redox status [21].

Defective cellular energy production seems to play an important role in the development of epilepsy, where mitochondrial dysfunction can induce cortical excitation of neurons. There is a complex interplay between oxidative stress, mitochondrial dysfunction and epileptogenesis. Inhibition of oxidative phosphorylation enzyme complexes in mitochondria result in decreased intracellular ATP levels. This in turn causes hyper-excitability of neurons by impairing sodium-potassium ATPase activity and decreasing membrane potential. Calcium sequestration, normally occurring in mitochondria, can no longer occur, rendering the neurons vulnerable to further excitotoxicity. This theory is backed by the fact that mutations in the mitochondrial respiratory chain complexes are known to result in neuronal hyperexcitability and epileptogenesis [22]. Experiments have also shown that the direct inhibition of enzymes in the mitochondrial respiratory chain using toxins can induce seizures in a dose-dependent manner [23]. Although dysfunction in any of the respiratory complexes can result in epileptogenesis, defects in complex **I** seems to be the most likely to result in seizures [2].

Hyper-excitability of neurons has also been proposed to be the result of excess glutamate release, which results from mitochondrial bioenergetics failure, dysfunction of the mitochondrial glutamate-aspartate transporter and loss of normal calcium signalling [2]. Excess release of glutamate has also been shown to have a direct effect on epileptogenesis and spread of epileptic activity across the cortex [24].

Furthermore, there is evidence to support that mitochondrial dysfunction can also play a role in seizure-related cell death. Kovac et al. [25] showed a direct association between blocking mitochondrial complex **I**, complex V or mitochondrial oxidative phosphorylation and rates of apoptosis, while conversely supplementing with the complex **I** substrate pyruvate led to decreased rates of apoptosis [25]. If the mitochondrial dysfunction can be prevented or treated, a level of neuroprotection would be expected, suggesting possible novel therapeutic avenues.

## 3. The Ketogenic Diet

Use of dietary manipulation and forms of fasting as a means of controlling epileptic seizures can be traced back to the time of Hippocrates, who was first to propose that seizures were not supernatural in origin [26]. In the 1920s, both in France and in the United States, significant discoveries were made regarding physiological changes linked with anti-seizure properties of fasting. The first to publish results showing the effectiveness of the ketogenic diet in epilepsy was Dr. Peterman, a paediatrician from the Mayo clinic in 1924. At the time the use of the KD was hyped due to limited alternatives, falling out of favour in the late 1930s due to its unpalatability and the emergence of AEDs like phenytoin [26,27,28]. 

The interest in the diet was re-ignited in the sixties due to growing evidence of its broad neuroprotective effects. The use of the KD is being studied in numerous other neurological diseases, which are felt to be arising partly from cellular energy failure and are characterized by neuronal death [18,27,28,29].

The KD is currently being used therapeutically for intractable epilepsy and for rare diseases of glucose metabolism, where it is the preferred first-line treatment. For instance, it is considered as the first-line treatment for glucose transporter type 1, pyruvate dehydrogenase deficiency and phosphofructokinase deficiency [17,30]. There is growing evidence to suggest that the KD should also be considered early in the treatment of other epilepsy syndromes like Dravet, West syndrome and myoclonic-astatic epilepsy (Doose syndrome) [27,30]. One variant of the KD, the low glycaemic index diet, has recently shown promise in the seizure control of patients with Angelman syndrome [31]. 

While there is now ample evidence of the benefits of the KD, one also needs to be aware of its limitations. The list of absolute contraindications includes fatty acid oxidation defects, pyruvate carboxylase deficiency and other gluconeogenesis defects, glycogen storage diseases, ketolysis defects, ketogenesis defects, porphyria, prolonged QT or other cardiac diseases, liver/kidney/or pancreatic insufficiency and hyperinsulinism. In addition, there are relative contraindications limiting its widespread use, like poor compliance, growth retardation, severe gastro-oesophageal reflux disease, inability to maintain adequate nutrition and a surgical focus identified by imaging or electroencephalogram [26,32].

The implementation of the KD can be arduous for families and requires close supervision by a specialized dietician, who will monitor the progress of the treatment and tailor it to each patient’s individual needs. This is especially important in the younger population where growth prevents protein intake to be below a certain minimum. Children under 6 months traditionally required an inpatient stay for the initiation of the treatment, although currently in the UK the aim is to start the KD on an outpatient basis.

Prior to initiation, baseline monitoring is advised by blood and urine tests along with measurement of growth parameters. During diet initiation monitoring occurs daily, so that changes can be made if side effects are intolerable. Side effects include gastrointestinal disturbance, impaired growth, hypoglycaemia, acidosis and dehydration at initiation, kidney stones, nutritional deficiencies, and hyperlipidaemia. Most of them can be alleviated by adjustments in the diet and vitamin, mineral and trace element supplementation [26,32]. Importantly, in the largest randomized trial to date the most frequent side-effects reported at 3-month review were constipation, vomiting, lack of energy, and hunger [33].

Finally, the KD therapy is not available in all epilepsy centres in the UK, despite evidence of its efficacy, due to funding restrictions and lack of resources, further limiting its use [27].

## 4. Mechanism of Action

**Figure 2:**
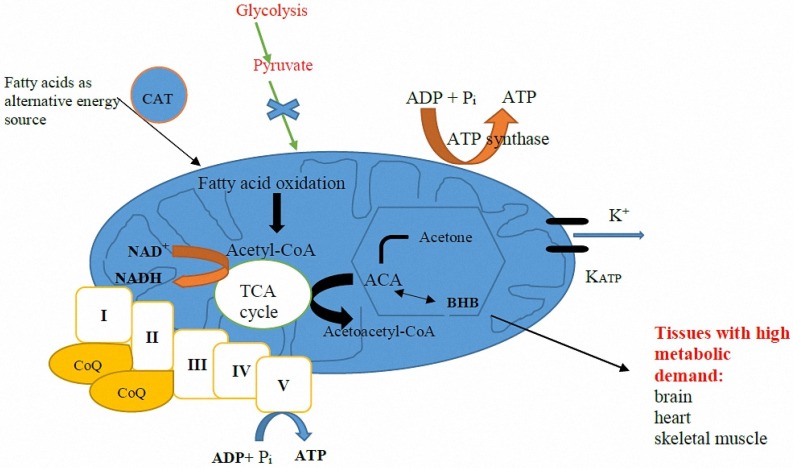
Mitochondri in hepatocyte. Ketone bodies alter mitochondrial metabolism at the cellular level; ketones reduce mitochondrial NAD coupling, oxidize Co-enzyme Q, increase the rate of ATP hydrolysis and increase metabolic efficiency. Oxidation of Co-enzyme Q decreases the major source reactive oxygen species (ROS). Increased rate of ATP hydrolysis widens the extra/intracellular ionic gradients, leading to hyperpolarization of cells. Potassium (K^+^): Hyperpolarization of cell membrane reducing excitotoxicity; CAT: carnitine-acylcarnitine translocase; BHB: β-hydroxybutyrate; ACA: acetoacetate; I–V: electron transport chain complexes; ADP: Adenosine Di-Phosphate; ATP: Adenosine Tri-Phosphate; CoQ10: Co-enzyme Q10; K_ATP_: ATP-sensitive potassium channels.

The KD is high in fat and low in carbohydrates, which mimics the metabolic state of starvation, forcing the body to utilize fat as its primary source of energy instead of carbohydrates. In the setting of elevated fatty acids and low dietary carbohydrate content while on a KD, the liver produces ketone bodies by shunting excess acetyl-CoA to ketogenesis (Figure 2). Thus, the two primary ketone bodies produced are acetoacetate and β-hydroxybutyrate (BHB). Acetoacetate metabolizes to acetone, the other major ketone elevated in patients on a KD.

However, for each of the proposed mechanisms in Table 1 below, there is evidence suggesting that it is unlikely to be the sole explanation for the efficacy of the ketogenic diet. It is more likely that the efficacy of the KD depends on many different mechanisms acting synergistically [28,34]. We will look at some of these theories in more detail.

Initially there was the theory that the antiepileptic effect of the KD was directly related to the ketosis, which we know now to be incorrect. BHB was thought to be the main ketone responsible for the anticonvulsant effect of the KD, with proposed mechanisms including reducing glycolysis, promoting endocytosis of synaptic vesicles more than exocytosis and conferring neuroprotection via hydroxylcarboxylic acid receptor 2 (HCA2) activation in macrophages [34]. Acetone was also shown to suppress acutely provoked seizures in animals, but the same has not been proven in vivo [28]. There is now plenty of evidence that shows poor correlation between plasma ketone levels and degree of seizure control [35,36].

Recent studies by Chang et al. [37] on hippocampal rat slices demonstrated that decanoic acid (CA10—a major component of the medium-chain fatty acid (MCT) diet), but not ketone bodies, has direct anti-epileptic properties at doses that produce similar plasma levels as those seen in patients on the MCT diet. Decanoic acid modifies the excitatory post-synaptic currents via direct inhibition of excitatory AMPA receptors. This effect is achieved through non-competitive binding to the M3 helix sites of the AMPA-GluA2 transmembrane domain. The antagonism of these AMPA receptors is essential in epilepsy treatment, as they are widespread throughout the brain, play a crucial role in generating and spreading epileptic activity and are implicated in seizure-induced cortical damage [38]. The co-administration of octanoic acid (CA8) enhances these effects and is independent of ketones bodies [39]. CA10 has also been associated with increased mitochondrial numbers, via the PPAR-γ receptor, and improved biogenesis, also suggesting that ketones bodies per se are not essential to bring about the anticonvulsant effects of the MCT diet [40]. Decanoic acid has also been shown to upregulate transcription of genes involved in fatty acid metabolism while down regulating those involved in glucose metabolism. Furthermore, medium-chain fatty acids have also been shown to modulate astrocyte metabolism (providing fuel in the form of lactate and ketones) and amino acid metabolism (increasing tryptophan), resulting in reduced neuronal excitability [34]. Various other medium chain triglycerides, like heptanoic acid and tridecanoin, are also being investigated for their anticonvulsant properties [34].

There is growing evidence that the KD alters the fundamental biochemistry of neurons in a manner that not only inhibits neuronal hyperexcitability, but also protects against a range of neurological disorders where cellular energy failure is felt to play a key role in pathogenesis [18,29]. In a rat kindling model, the KD was shown to reduce oxidative stress, reduce glycolysis, diminish spontaneous firing of ATP-sensitive potassium channels and delay epileptogenesis [41]. The KD has been shown to be neuroprotective in animal models of several CNS disorders, including Alzheimer’s disease, Parkinson’s disease, hypoxia, glutamate toxicity, ischemia, and traumatic brain injury. This neuroprotective effect may be possible through antioxidant and anti-inflammatory actions [29]. This is supported by studies in animal models and isolated cells that showed neuroprotection against many types of cellular injury is afforded by ketone bodies, especially BHB [29]. Thus, the KD may ultimately prove useful in the treatment of a variety of neurological disorders [18,41].

While initially there were concerns about KD initiation in the younger population due to its possible side-effects on growth, the KD has been proven to be very effective in this age group. Its efficacy can be partly explained by the higher levels of ketone metabolizing enzymes and monocarboxylic acid transporters produced during infancy, which transfer ketone bodies across the blood-brain barrier [18]. Thus, the brain is much more efficient at extracting and utilizing ketone bodies from the blood in this age group.

Current evidence suggests that a fundamental shift from glycolysis to intermediary metabolism induced by the KD is both necessary and sufficient for clinical efficacy. This notion is supported by a growing number of studies indicating that metabolic changes likely related to the KD’s anticonvulsant properties include ketosis, reduced glycolysis, protein restriction, elevated fatty acid levels, and enhanced bioenergetic reserves [34,41,42]. Direct neuronal effects induced by the diet may involve ATP-sensitive potassium channel modulation, enhanced purinergic (i.e., adenosine) and GABAergic (gabba-aminobutyric acid) neurotransmission, increased brain-derived neurotrophic factor (BDNF) expression, attenuation of neuroinflammation, and expansion in energy reserves and stabilization of the neuronal membrane potential through improved mitochondrial function [43]. 

The observation that the full anticonvulsant effect of the KD can take weeks to develop, suggested that altered gene expression could be implicated [44]. It has been proposed that the KD may increase mitochondrial biogenesis, by inducing transcription of some electron transport chain subunit messenger ribonucleic acids (mRNAs) [44]. This results in increased ATP levels, leading to increased neuronal “energy reserves”, allowing neurons to withstand metabolic challenges and stabilize neuronal membrane potential [28,29,44]. There is a theory that suggests that the disease-modifying effects of the KD on epilepsy are exerted via an adenosine-dependent epigenetic mechanism [34].

Ketone bodies may alter the behaviour of vesicular glutamate transporters (VGLUTs) responsible for filling pre-synaptic vesicles with glutamate. In Juge et al. ketone bodies were shown to inhibit glutamate release by competing with Cl^−^ at the site of VGLUT regulation [45]. 

Potassium-ATP channels are of particular interest given their close relationship with cellular metabolism. Their hyperpolarization was shown to have seizure suppressing effects and they were seen to increase in numbers when ATP was low [46]. Ketones have been shown to exert their neuroprotective and anticonvulsant effects by genetically modulating potassium-ATP channels [47].

## 5. Different Variants of the Ketogenic Diet

There are five main categories of ketogenic diets, initially proposed in 1921 [18].

The first is the classic KD, which uses a 4:1 or 3:1 fat-to-carbohydrate and protein ratio. This means that about 90% of a daily caloric intake comes from fat and the rest includes a small amount of protein, which ensures adequate growth, especially important in the paediatric population. The ratio can be altered to 2.5:1, 2:1 or 1:1 based on the patient’s needs. A dietician will implement and closely monitor the diet and tailor it accordingly. This type of diet uses saturated long-chain fatty acids as the main source of energy [18,27].

Seizure control on the classic KD slowly increases within days to weeks of starting the diet. However, the serum levels of the ketone bodies do not correlate tightly with seizure control. The mechanism of their anti-seizure properties remains unclear. Interestingly, the ingestion of carbohydrates more than what is allowed rapidly reverses the therapeutic effect. Seizures can start within minutes of ingesting carbohydrates. 

The second is the MCT diet, which was introduced in 1971 to surpass the severe restrictions of the classic KD and to make it more palatable. The main fatty acids used are caprylic acid (CA8), capric acid (CA10) and, to a lesser extent, caproic acid and lauric acid. This diet is not based on diet ratios, but uses a percentage of calories from MCT oils to create ketones. The main advantage of this diet over long-chain triglycerides (LCTs) is that MCTs are quickly transported to the liver by albumin and are more efficiently absorbed across mitochondrial membranes, without binding on carnitine [28]. Thus, MCT metabolism is faster, requires less energy expenditure, produces a higher level of ketosis than LCTs and is more palatable, as less total fat is required and more protein and carbohydrates can be consumed.

The trial designed to compare the efficacy of this diet with the classic KD in children with intractable epilepsy at 3, 6 and 12 months, found no differences between the dietary groups in the percent reduction of seizures. Importantly, there was no difference in the percentages of children achieving reduction in seizures greater than 50% or 90% [48]. The authors concluded that the diets were comparable in efficacy and tolerability, which offers a degree of choice to the patient and their family as to which diet they will use [27,47]. Chang et al. compared the effects of several MCTs with valproic acid both in vivo and in vitro and found that decanoic acid specifically was superior in controlling seizures and had fewer adverse side effects [49].

The third is the modified Atkins diet, which was originally designed and investigated at Johns Hopkins Hospital [50] and proposed as a less restrictive and more palatable dietary treatment. Contrary to other KDs, it does not overly restrict protein intake or daily calories to make it more palatable and increases compliance, especially in adults [51]. This diet restricts carbohydrates to 10 g/day for children (15 g/day in adults) while encouraging high-fat foods [52,53]. There is scope for modifying the carbohydrate content depending on seizure control [18].

There have been about 400 children and adults on the modified Atkins diet, as treatment for intractable epilepsy, taking part in over thirty prospective and retrospective studies published worldwide in the past 10 years. The studies have consistently shown similar efficacy of the modified Atkins diet to the classical KD and improved tolerability. Further studies would clarify its role in the management of intractable epilepsy and in other neurological disorders [52,53].

The fourth is the low glycaemic index diet, which allows a more moderate intake of carbohydrates, as long as they have a glycaemic index lower than 50, without increasing ketone levels [54]. The diet was implemented based on clinical observation that preventing large postprandial increases in blood glucose and maintaining as stable as possible blood glucose levels resulted in improved seizure control in a broad range of patients [51]. 

In a study of 20 paediatric and adult patients treated with the low glycaemic index diet, 50% experienced a greater than 90% reduction in seizure frequency, despite achieving lower blood levels of ketone bodies [51]. In another study where 89% of patients had not responded to 3 AEDs, 76 children were treated with this diet and followed up for up to 1 year. There was a greater than 50% seizure reduction in 66% of patients at 12 months. The study also showed an inverse relationship between efficacy of the diet and serum glucose, but did not find a correlation between seizure reduction and circulating levels of BHB. The commonest reason for discontinuing the diet was diet restrictiveness [55].

Lastly, the fifth is the calorie restriction or intermittent fasting diet, which involves the reduction in total caloric intake without a risk of malnutrition. No clinical studies exist yet which assess its effect on seizure suppression. Some authors believe that it is the restriction of calories, not the composition of the diet per se, that has the anti-convulsive effects, but this remains to be proven [18].

## 6. Evidence: Use of the Ketogenic Diet in Intractable Epilepsy in Mitochondrial Disorders

The use of the KD has been proven to be a safe and effective treatment in intractable epilepsy in numerous studies [42]. However, its specific use in mitochondrial disorders has not been extensively studies yet. The evidence of KD benefits and in the management of mitochondrial diseases, and specifically in the treatment of seizure disorders due to either nuclear or mitochondrial DNA defects is growing [11,13,56,57]. The efficacy of the KD has also been investigated in a variety of other CNS disorders, including Alzheimer’s disease (AD), Parkinson’s disease, headache, hypoxia, glutamate toxicity, autism, ischemia, and traumatic brain injury [18].

Jarrett et al. [58] demonstrated that the KD confers protection to the mitochondrial genome against oxidative insults, increasing the levels and stimulating de novo biosynthesis of mitochondrial glutathione and improving mitochondrial redox status. This results in improved cellular metabolism and may explain the KD’s efficacy in mitochondrial diseases.

Lee et al. [13] looked at 48 patients with confirmed mitochondrial respiratory chain defects and epilepsy. Out of all the patients with medically intractable epilepsy (four or more seizures per month, resistant to three or more antiepileptic drugs), they treated 24 children with the classic ketogenic diet (4:1) and assessed its efficacy. They found that 75% of individuals had seizure reduction of more than 50%, and half of people became seizure free [13]. However, of the 31 children (64.5%) with medically intractable epilepsy only 24 received the ketogenic diet, without explaining what happened to the other 7. Furthermore, three (12.5%) patients had to stop the ketogenic diet due to serious infections or persistent metabolic acidosis.

In the retrospective study, Kang H.C. et al. [57] evaluated the clinical efficacy and safety of the classic KD for 14 patients (1.7–11.8 years of age) with intractable epilepsy and mitochondrial respiratory chain complex defects. Seven patients (50%) became seizure-free after KD initiation, three of whom successfully completed the diet without relapse. One patient had a greater than 90% seizure reduction, and two patients with a seizure reduction between 50% and 90%, remained on the diet. They concluded that the KD was a safe and effective therapy for seizures in children with intractable epilepsy and respiratory chain complex defects.

Furthermore, the KD was also reported to be a useful adjunct to traditional pharmaceutical agents. In Martikainen et al., a low glycaemic index diet was used in conjunction with three anti-epileptic drugs in a 26-year-old woman and was found to be effective and well tolerated as treatment for severe episodes of POLG-related mitochondrial epilepsy [56]. Furthermore, in a case of Ohtahara syndrome with mitochondrial respiratory chain complex **I** deficiency the seizures stopped and the burst suppressions pattern disappeared following 3 months on the KD therapy with vitamins, coenzyme therapy and antioxidant treatment [59]. Also, a five-year-old female with Landau-Kleffner and respiratory chain complex **I** deficiency was successfully treated with the KD in combination with coenzyme Q10, riboflavin, l-carnitine, and high-dose multivitamins [60]. However, these are single cases and lack statistical power.

In Barnerias et al., they studied 22 patients (early infancy to 30 years old), with confirmed pyruvate dehydrogenase complex deficiency [61]. Nine out of the 22 (41%) had epilepsy as one of the clinical manifestations. Of those treated with the KD, five showed clear benefit on childhood-onset epilepsy (two patients) or paroxysmal dystonia (three patients). This study showed that the KD was more efficacious against paroxysmal energy dysfunction seen in late-onset epilepsy and paroxysmal dystonia or ataxia than early onset epilepsy associated with infantile spasms [61].

In a randomized controlled trial over 5 years, Neal tested the efficacy of the KD given for 3 months on children aged 2–16 years with refractory epilepsy of different aetiologies [33]. Children were included if they had either daily seizures or at least seven seizures per week and had failed therapy with at least two AEDs. Twenty-eight children (38%) on the diet had greater than 50% seizure reduction compared with four (6%) controls (*p* < 0.0001), and five children (7%) on the diet had greater than 90% seizure reduction compared with none in the control group. The results favoured the use of the KD in intractable epilepsy with efficacy rates comparable to that of new AEDs [33]. Although this trial provided definitive evidence of the efficacy of the KD in intractable epilepsy, it did not look specifically at epilepsy in the context of mitochondrial disorders. 

It has also been demonstrated that children benefitting on the KD show an improved neurodevelopmental outcome as early as three months after diet initiation [62]. However, Dressler et al. did not utilize a formal neuropsychological assessment in their study [62]. Instead, the improved psychomotor development was based on clinical neurological examination and EEG. In Zhu et al. [63], assessment using Gesell developmental scales assessment revealed improved neurodevelopmental progress in 42 children (6 months to 6 years of age) treated with the classic KD for intractable epilepsy. They were assessed at 3, 6, 12 and 18 months after starting the KD. They compared their data with those before the KD treatment and found statistically significant differences. It was concluded that the improvement in neurobehavioral outcome was dependent on clinical seizure control and the benefit was more pronounced with prolonged length of treatment with the KD [63]. Of note, both these studies included children with intractable epilepsy due to a variety of aetiologies and not specifically mitochondrial disorders. More research is needed to determine the effect of the KD on neurodevelopment in children with mitochondrial disorders and epilepsy.

## 7. Conclusions

There is growing evidence of the benefits of the KD and its variants in the treatment of a variety of neurological and neurodegenerative disorders. This suggests a possible common central mechanism that improves cellular metabolism and allows cells to resist metabolic challenges that lead to apoptosis. Identification of a correlation between caloric restriction with ketosis and protection from oxidative stress could reveal new therapeutic paths in age-related neurological disorders, like Alzheimer’s. Although the full mechanism by which the KD and its variants improve oncological and neurological conditions remains to be elucidated, their clinical efficacy has attracted many new followers, and has re-ignited research interest in this field [27,28,29,54]. The epigenetic mechanism of action of the KD means it can alter the course of the epilepsy [1], and may explain why its anticonvulsant effects are long-lasting, even after the KD is stopped.

Further understanding of how this diet exerts its effects would allow its use to become broader and perhaps the development of a more palatable and less strict diet regimen. Research interest is also centred around the possibility of replacing the strict diet with its challenging adherence rules with supplements, like a “ketogenic pill”. Ketone esters given orally were effective anticonvulsants in mouse models of Angelmans and in pentylenetetrazole-model of seizures [64,65]. In addition, in Sada et al., lactate dehydrogensae (LDH) inhibition was seen to be effective in suppressing seizures. The inhibition of this metabolic pathway was seen to mimic the KD effects, providing further hope that one day a pill may be devised that will be able to provide the benefits of the KD without the strict adherence rules [66]. In the future, novel antiepileptic drugs may contain antioxidant properties that target neurodegeneration and mimic the effects of the KD [19].

The safety of the diet also needs to be investigated further in the younger population as the randomized controlled study by Neal et al. only looked at children older than 2 years [33]. More work needs to be done to establish which patients would benefit the most, especially when the diet is being considered for a wider range of neurological disorders.

Although there is a significant body of evidence that supports the use of the KD in intractable epilepsy, more studies need to be done looking specifically at its effect in intractable epilepsy in the context of mitochondrial disorders.

## Figures and Tables

**Table 1:** Proposed mechanisms of action of the ketogenic diet (KD).

1. Anti-inflammatory action and neuroprotection against excitotoxicity and oxidative stress (enhanced bioenergetics reserves, antioxidant mechanisms).
2. Direct anticonvulsant action of ketones (e.g., reduces hyperexcitability, supports synaptic vesicle recycling, inhibits glutamate release).
3. Direct anticonvulsant action of polyunsaturated fatty acids and protein and glycolytic restriction.
4. Modulation of sodium and potassium and calcium balance (e.g., opening of voltage-gated potassium channels via poly-unsaturated fatty acids, ketone modulation of ATP-sensitive potassium channels, blockage of voltage-gated calcium channels).
5. Direct anticonvulsant action of medium-chain fatty acids (MCTs) (e.g., decanoic acid (CA10), direct inhibition of a-amino-3-hydroxy-5-methyl-4-isoxazolepropionic acid receptor (AMPA) receptors, MCT modulation of amino acid metabolism).
6. Disease-modifying anticonvulsant effects/epigenetic mechanisms (e.g., upregulation of transcription of genes involved in fatty acid metabolism while down regulating those involved in glucose metabolism, improved mitochondrial biogenesis and increase in mitochondrial numbers via peroxisome proliferator-activated receptor gamma (PPAR-γ), MCT modulation of astrocyte metabolism).
